# Progress in Surgery

**Published:** 1895-04-13

**Authors:** 


### PROGRESS IN SURGERY.

### GENITO-URINARY SURGERY.

(*Continued from page* 11.)

**The Surgery of the Ureter.**—A very complete account of recent work in connection with the surgery of the ureter is given by Dr. Christian Fenger, of Chicago.[9] The first question discussed is how best to reach the ureter. The entire length of the ureter can be reached by the median or lateral abdominal incision, and this method is of much value for exploration in cases of suspected calculus in the ureter or a collection of urine following rupture, but it is a dangerous method of opening the ureter, because of the risk of infection of the peritoneum by the escape of possibly septic urine into the peritoneum. Hence, if employed for exploration, the actual opening of the ureter should be done by means of an extra-peritoneal incision. Extra-peritoneal access to the upper two-thirds of the ureter can be obtained by a prolongation downwards of the nephrotomy incision. The ordinary oblique incision is prolonged downwards an inch anterior to the ilium and along Poupart's ligament to about its middle. Cotterell[10] has pointed out that the middle three-fifths of the ureter may be explored by an incision similar to that for ligature of the common iliac artery. The pelvic portion of the ureter can be reached in women through the vagina, but it is very difficult to reach this portion of the ureter in males. It is proposed to get at it by an operation similar to Kraske's for excision of the rectum high up. Cotterell[11] would prefer a perineal operation, dissecting down between the urethra and rectum to the base of the bladder. The difficulty of dealing with ruptures of the ureter lies in the fact that it is almost impossible to diagnose them at the time of the injury. Hæmaturia may be very slight and transient, or entirely absent. Some days or weeks after the rupture a round or sausage-shaped swelling forms in the course of the canal, and is palpable from the abdomen. It is usually painful. Most cases have been treated by repeated punctures, or incision and drainage, and when the latter method has been adopted septic infection of the kidney has necessitated secondary nephrotomy. Whether in these cases the ureter can be found in the cavity which usually contains infected urine, and the ruptured ends reunited, has yet to be decided by experience. If only the condition could be diagnosed at an earlier period this could almost certainly be done.

When a calculus is impacted in the ureter it is impossible to be certain of its locality, unless it can be felt in the lower end of the ureter either from the rectum or vagina. The symptoms of impacted calculus are referred to in The Hospital, December 29th, 1894, p. 223, and the methods of reaching the ureter for removal of the calculus have already been described. It is not necessary to suture the wound in the ureter (which should be a longitudinal one), unless the stone is removed from the abdomen through the peritoneal cavity, but, as before stated, this is a dangerous method, and should not be employed.

In cases of hydronephrosis or pyonephrosis the ureter should be explored in its entire length by means of a long flexible silver probe or an elastic bougie of 9 to 12 French scale passed from the opening into the kidney pelvis. If the opening of the ureter cannot be found in the kidney pelvis, the ureter must be opened. If valve formation at the commencement of the ureter is the cause of the obstruction, the valve should be split longitudinally, and the flaps thus formed turned out and united to the inner wall of the sac by sutures. Strictures should be divided by longitudinal incision, and the edges of the incision then sewn together in a transverse direction, the tube being thus bent at an angle. If the ureter is long enough to allow of excision of the strictured portion, and implantation of the upper end into the lower in the way presently to be described, that would be the preferable method.

The ureters can be catheterised from the bladder without great difficulty in the female, and thus the diagnosis of renal affections facilitated, and even strictures of the ureter have been treated in this way. In males, catheterisation of the ureter can only be done after suprapubic cystotomy, but may have an occasional use in connection with an exploratory operation of this nature.

The ureter may be wounded in hysterectomy or the removal of ovarian tumours, particularly those in the broad ligament. An intra-peritoneal longitudinal wound should be carefully united with extra mucous sutures, and a fold of peritoneum slid from both sides over the sutured wound, or an omental graft employed. Merely sewing together transverse wounds of the ureter has not been satisfactory, and the junction usually fails, or, when it does not, serious stenosis or complete obliteration of the canal may take place. The best method for the union of a transversely divided ureter seems to be Van Hook's, which consists of an implantation of the proximal end into the distal through a longitudinal slit in its wall, after ligature of its cut end. It resembles an intussusception. For the details reference must be made to Fenger's paper or Van Hook's.[12] This junction of the divided ureter has received the name of uretero-ureterostomy. If there has been so great a loss of substance as to make re-union of the two ends impossible, and the upper end of the divided ureter is long enough to reach the bladder it should be implanted into it. Cases of uretero-uterine or uretero-vaginal fistulæ have been treated by vaginal plastic operations for displacement of the proximal end of the ureter into the bladder. Repeated attempts at closure of the fistulæ have often to be made, and in many the kidney has been removed as a last resource. Fenger thinks that the method of implantation into the bladder is the operation of the future. Whenever possible, the ureter, either after loss of substance or in fistulæ, should be implanted into the bladder. Experiments show that if the ureter is implanted into the bowel—as might be expected—septic infection of the kidney is liable to occur, and the same must be said of implantation on to the skin, though here the risk is not so great.

[9] Annals of Surgery, Sept. 1894, p. 257. [10] Lancet, April 14, 1894. [11] Soc. Cit. [12] Journal American Medical Association, Dec. 16, 1893.

